# Metabolic regulation of the mitochondrial immune checkpoint

**DOI:** 10.1080/2162402X.2024.2394247

**Published:** 2024-08-26

**Authors:** David C. Montrose, Suchandrima Saha, Lorenzo Galluzzi

**Affiliations:** a Department of Pathology, Renaissance School of Medicine, Stony Brook University, Stony Brook, NY, USA; b Stony Brook Cancer Center, Stony Brook, NY, USA; c Department of Radiation Oncology, Weill Cornell Medicine, New York, NY, USA; d Sandra and Edward Meyer Cancer Center, New York, NY, USA; e Caryl and Israel Englander Institute for Precision Medicine, New York, NY, USA

**Keywords:** Apoptotic caspases, BCL2, immune checkpoint inhibition, MOMP, senescence, type I interferon

## Abstract

Disrupting mitochondrial function in malignant cells is a promising strategy to enhance anticancer immunity. We have recently demonstrated that depriving colorectal cancer cells of serine results in mitochondrial dysfunction coupled with the cytosolic accumulation of mitochondrial DNA and consequent activation of CGAS- and STING-dependent tumor-targeting immune responses.

## Main text

A variety of tumors exhibit defects that directly or indirectly interfere with the molecular machinery responsible for so-called “mitochondrial outer membrane permeabilization” (MOMP), a critical step in apoptotic cell death.^1^ Such defects encompass loss-of-function mutations in genes coding for MOMP enablers, such as tumor protein p53 (*TP53*); amplifications in genes encoding MOMP inhibitors, notably BCL2 apoptosis regulator (*BCL2*); and a number of epigenetic alterations affecting the expression levels of numerous MOMP regulators.^2^ Along similar lines, multiple tumors are characterized by an increased threshold for the so-called “mitochondrial permeability transition” (MPT), a critical step in a specific form of regulated necrosis that involves a rapid loss of ionic barrier functions of the inner mitochondrial membrane.^3^

For many years, it was believed that these alterations only serve as means for malignant cells to resist cell death and proliferate despite harsh microenvironmental conditions, which emerge naturally in the tumor microenvironment or are elicited by therapy.^4^ Importantly, it is now clear that both normal and malignant cells also harness MOMP- and MPT-inhibitory mechanisms, as well as MOMP-compensatory pathways such as apoptotic caspase activation, to prevent the initiation of unwarranted inflammatory responses that – at least in some oncological settings – may (re)instate immune disease control.^5^ Mitochondria indeed contain a number of damage-associated molecular patterns (DAMPs) that – once released in the cytosol or the extracellular milieu – can potently promote inflammation, such as mitochondrial DNA (mtDNA).^5^ The cytosolic accumulation of mtDNA promotes the cyclic GMP-AMP synthase (CGAS)-dependent activation of the stimulator of interferon response cGAMP interactor 1 (STING1, best known as STING), culminating in inflammatory signaling *via* NF-κB and interferon regulatory factor 3 (IRF3).^5^ Corroborating the importance of this pathway for cancer immune evasion, genetic and pharmacological strategies enabling MOMP and/or MPT and inhibiting apoptotic caspases have been shown to promote type I IFN coupled with immunological tumor control – be it natural or elicited by (immuno)therapy – in a variety of preclinical cancer models.^6–8^ We have recently demonstrated that the non-essential amino acid serine is key for the preservation of a such a mitochondrial immune checkpoint in colorectal cancer (CRC) cells.^9^

We investigated the mitochondrial effects of the depletion of both endogenous and exogenous serine by utilizing mouse CRC CT26 cells defective for phosphoserine aminotransferase 1 (PSAT1) – an essential component of the serine synthesis pathway,^10^ cultured in a serine-replete or deplete medium. As compared to control conditions as well as to the restriction of either endogenous or exogenous serine, complete serine deprivation promoted the loss of mitochondrial transmembrane potential and overgeneration of mitochondrial reactive oxygen species (ROS), coupled with maximally reduced ATP production and cytosolic accumulation of double stranded DNA (dsDNA). Corroborating the mitochondrial origin of the latter: (1) dsDNA species accumulating in the cytosol of serine-restricted CT26 cells mostly colocalized with two distinct mitochondrial proteins (rather than with a nuclear marker), and (2) cytosolic dsDNA buildup could be prevented by mtDNA depletion or pharmacological inhibition of MPT with cyclosporin A (CsA).^9^

We next tested whether mtDNA release, as observed in serine-deprived CT26 cells, effectively initiates CGAS-STING signaling. Indeed, serine restriction elicited increased intracellular levels of the CGAS-derived STING activator cyclic GMP-AMP (cGAMP) as well as (1) the activating phosphorylation of multiple STING signal transducers including IRF3 and (2) the upregulation of multiple IRF3 target genes including interferon beta 1 (*Ifnb1*) and consequent IFNB1 secretion. Importantly, all these manifestations of active STING signaling in serine-depleted CT26 cells could be prevented by mtDNA depletion or CsA administration.^9^

PSAT1-deficient CT26 tumors established in immunocompetent syngeneic BALB/c mice receiving a serine-deprived diet exhibited a growth disadvantage as compared to their control counterparts (irrespective of mouse diet) as well as compared to PSAT1-defective CT26 tumors established in BALB/c mice receiving a control diet. This was paralleled by the intratumoral upregulation of multiple IRF3 targets coupled with the recruitment of mature dendritic cells (DCs) as well as CD8^+^ and CD4^+^ T cells. Importantly, blocking interferon alpha and beta receptor subunit 1 (IFNAR1) or co-depleting CD8^+^ and CD4^+^ T cells was sufficient to abrogate the growth disadvantage of PSAT1-deficient CT26 tumors established in immunocompetent syngeneic BALB/c mice under dietary serine restriction. Notably, tumor growth suppression and the associated recruitment of mature DCs as well as CD8^+^ and CD4^+^ T cells caused by exogenous serine restriction in PSAT1-defective CT26 lesions could also be abrogated by the shRNA-mediated depletion of STING. Taken together, these findings mechanistically connect serine depletion with STING-dependent and type I IFN-driven anticancer immunity.^9^

Immunophenotyping serine-deprived CT26 tumors revealed that tumor-infiltrating CD8^+^ T cells exhibit multiple markers of terminal dysfunction, notably programmed cell death 1 (PDCD1, best known as PD-1), pointing to the possibility of harnessing a PD-1 blocker to further increase disease control. Indeed, targeting PD-1 was considerably more effective against CT26 tumors in the context of endogenous and exogenous serine restriction, leading to complete disease eradication in approximately 45% of mice. Corroborating the establishment of immunological memory, animals that experienced tumor eradication were protected against a rechallenge with live PSAT1-defective CT26 cells while allowing for the development of mouse mammary carcinoma 4T1 lesions.^9^

**Figure 1.**
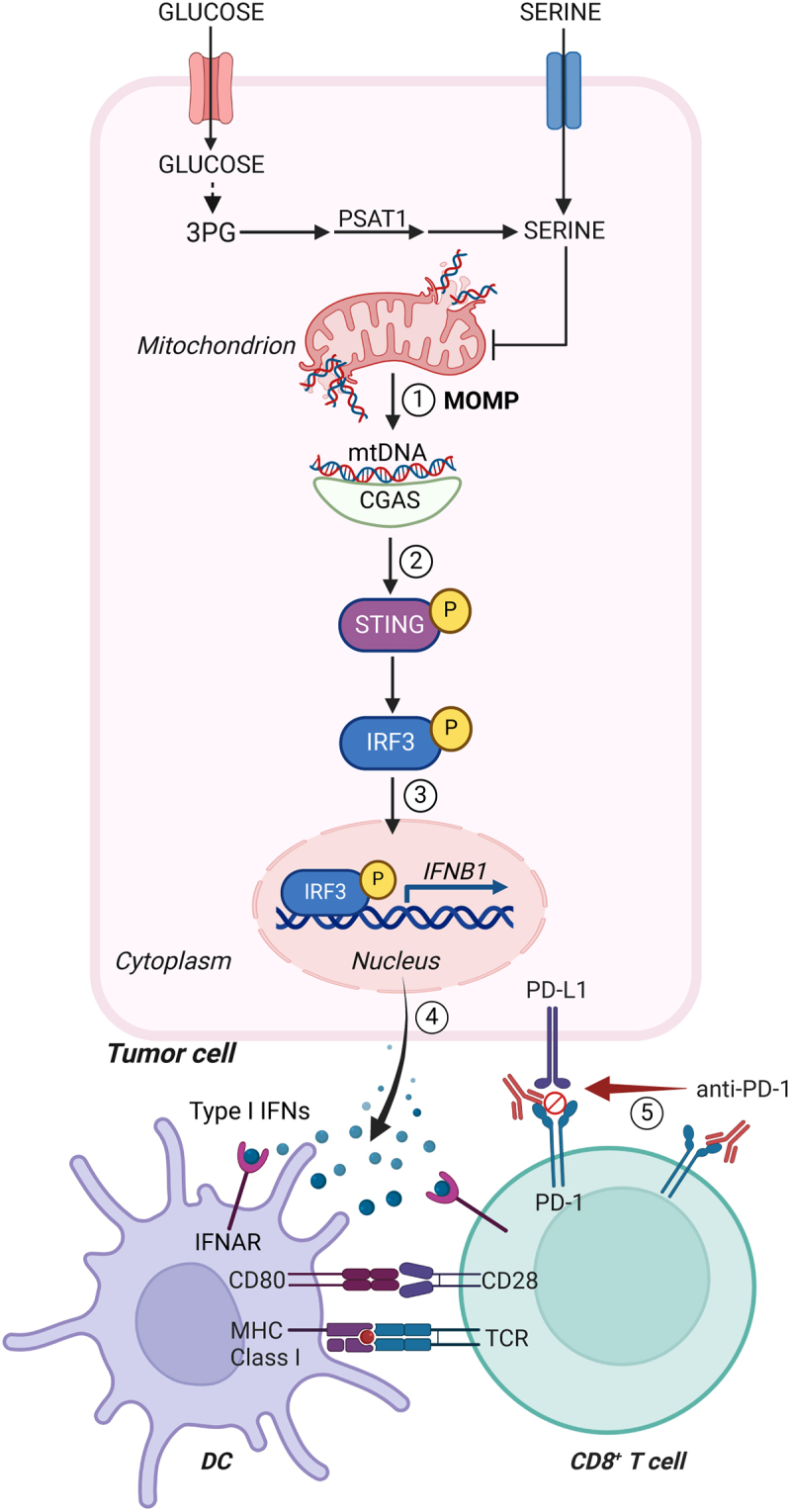
Serine-dependent control of the mitochondrial immune checkpoint. Restricting both endogenous and exogenous sources of serine in colorectal cancer cells promotes mitochondrial dysfunction coupled with (1) cytosolic accumulation of mitochondrial DNA (mtDNA); (2) cyclic GMP-AMP synthase (CGAS)-dependent activation of stimulator of interferon response cGAMP interactor 1 (STING1, best known as STING); (3) interferon regulatory factor 3 (IRF3)-dependent upregulation of interferon-stimulated genes (ISGs), including interferon beta 1 (*IFNB1*); (4) recruitment of mature dendritic cells (DCs) as well as CD4^+^ and CD8^+^ T cells; and (5) initiation of an anticancer immune response that can be actioned by programmed cell death 1 (PDCD1, best known as PD-1) blockers. 3PG, 3-phosphoglyceric acid; DC, dendritic cell; IFN, interferon; IFNAR, interferon (alpha and beta) receptor; MOMP, mitochondrial outer membrane permeabilization; PD-L1 (official name CD274); PSAT1, phosphoserine aminotransferase 1; TCR, T cell receptor.

Taken together, our findings demonstrate that restricting both endogenous and exogenous serine to CRC cells results in abundant mtDNA-driven, CGAS- and STING-dependent secretion of type I IFN along with restored tumor sensitivity to PD-1 blockage, thereby delineating a novel metabolic mechanism though which malignant cells preserve the mitochondrial immune checkpoint (Figure 1). Systemic nutritional interventions targeting both endogenous and exogenous sources of serine may be difficult to implement in the clinic, at least in part reflecting the notion that serine is highly required for normal cells, not only as a protein building block but also as a metabolic intermediate.^10^ That said, our findings suggest that detecting a naturally reduced metabolic flux through serine synthesis may be useful to identify patients with microsatellite stable CRCs who would benefit from ICIs, either as standalone interventions or coupled with dietary serine restriction. Supporting this possibility, our bioinformatic analysis suggests a negative correlation between predicted serine levels and anticancer immunity in human CRC.^9^ Clinical trials investigating this prospect are urgently awaited.
